# Can using the functional resonance analysis method, as an intervention, improve patient safety in hospitals?: a stepped wedge design protocol

**DOI:** 10.1186/s12913-021-07244-z

**Published:** 2021-11-13

**Authors:** Liselotte M. van Dijk, Meggie D. Meulman, Linda van Eikenhorst, Hanneke Merten, Bernadette C. F. M. Schutijser, Cordula Wagner

**Affiliations:** 1 Netherlands Institute for Health Services Research (Nivel), PO Box 1568, 3500 Utrecht, BN Netherlands; 2 Department of Public and Occupational Health, Amsterdam Public Health Research Institute, Amsterdam UMC, Vrije Universiteit Amsterdam, De Boelelaan, 1117 Amsterdam, Netherlands

**Keywords:** Patient safety, Stepped wedge trial, FRAM, Safety-II, Hospital

## Abstract

**Background:**

Healthcare professionals are sometimes forced to adjust their work to varying conditions leading to discrepancies between hospital protocols and daily practice. We will examine the discrepancies between protocols, ‘Work As Imagined’ (WAI), and daily practice ‘Work As Done’ (WAD) to determine whether these adjustments are deliberate or accidental. The discrepancies between WAI and WAD can be visualised using the Functional Resonance Analysis Method (FRAM). FRAM will be applied to three patient safety themes: risk screening of the frail older patients; the administration of high-risk medication; and performing medication reconciliation at discharge.

**Methods:**

A stepped wedge design will be used to collect data over 16 months. The FRAM intervention consists of constructing WAI and WAD models by analysing hospital protocols and interviewing healthcare professionals, and a meeting with healthcare professionals in each ward to discuss the discrepancies between WAI and WAD. Safety indicators will be collected to monitor compliance rates. Additionally, the potential differences in resilience levels among nurses before and after the FRAM intervention will be measured using the Employee Resilience Scale (EmpRes) questionnaire. Lastly, we will monitor whether gaining insight into differences between WAI and WAD has led to behavioural and organisational change.

**Discussion:**

This article will assess whether using FRAM to reveal possible discrepancies between hospital protocols (WAI) and daily practice (WAD) will improve compliance with safety indicators and employee resilience, and whether these insights will lead to behavioural and organisational change.

**Trial registration:**

Netherlands Trial Register NL8778; https://www.trialregister.nl/trial/8778. Registered 16 July 2020. Retrospectively registered.

**Supplementary Information:**

The online version contains supplementary material available at 10.1186/s12913-021-07244-z.

## Background

In the past years, several countries have launched safety campaigns and programmes to improve patient safety [1–6]. The World Health Organisation (WHO) defines patient safety as: ‘*The absence of preventable harm to a patient during the process of healthcare and reduction of risk of unnecessary harm associated with health care to an acceptable minimum*’ [7]. Consequently, several countries assessed how many patients were harmed during healthcare by examining the nature and extent of adverse events (AEs) in their hospitals [1, 2, 8]. AEs are defined as unintended injuries, resulting in a temporary or permanent disability, death or prolonged hospital stay caused by healthcare management [9, 10].

In the Netherlands, the incidence of such events in hospitals was first assessed in 2004 [1]. This study helped to identify ten themes eligible for improvement of patient safety, including the themes risk screening of frail older patients, administration of high-risk medication such as intravenous infusion and subcutaneous or intramuscular injections, and medication reconciliation at discharge. Subsequently, the Dutch Safety Management Programme (VMS in Dutch abbreviation) was developed to reduce risks for patients and to make healthcare processes safer [3, 11–14]. The program ran from 2008 to 2012 and led to a significant reduction of preventable AEs (from 2.3 to 1.6%) [3, 15]. In the following years after the safety programme, the incidence rate of AEs further decreased from 11.9% in 2012 to 9.9% in 2016. However, it did not reveal a further reduction of potentially preventable AEs (4.0% in 2012 to 4.3% in 2016) nor potentially preventable deaths related to AEs (2.6% in 2012 to 3,1% in 2016) [3, 16].

Even though the first studies showed improvements on the incidence rates of AEs, including preventable ones, the results from several countries are stagnating [17–19]. This raises doubt on whether existing improvement initiatives are still sufficient, as they mainly focus on the implementation of guidelines and protocols. This has resulted in a shift in the approach of patient safety [17]. Previously, patient safety was approached from the ‘Safety-I’ perspective, assessing safety as the absence of adverse outcomes by human or system failures [20]. Over the years, identification of human or system failures has resulted in adjustments in standard procedures. However, if existing processes are not taken into account, this could result in complex and unworkable procedures [20–22]. Protocols describe how care processes should be executed and often such processes are seen as linear. In practice, these processes are subjected to day to day variability of providing care. Different processes often occur simultaneously or are intertwined. This leads to more dynamic and complex care processes than imagined in a protocol [20–24]. Achieving absolute compliance with guidelines and protocols may be too ambitious, as daily practice requires adaptations to changing work conditions and constraints upon resources [20]. For that reason, the interest in the ‘Safety-II’ perspective is increasing, as it seeks to understand daily practice better and focusses on why things often go right [20, 25]. Within this perspective, practice variation is not perceived as a ‘negative’ factor which must be restrained by standardisation, but as a logical consequence of the need to adapt in order to succeed despite changing circumstances [20, 22, 25]. Healthcare professionals need to adjust their work to varying conditions (i.e. being resilient) [22, 26–28]. However, this adaptation could lead to discrepancies between daily practice (Work-As-Done (WAD)) and how the process is described in protocols (Work-As-Imagined (WAI)). The Functional Resonance Analysis Method (FRAM) originates from the Safety-II perspective and can characterise the potential variability of functions caused by resilience shown by healthcare professionals [29]. All activities within care processes can be visualized using the FRAM [20]. This can offer opportunities for education about organisational factors [24]. Understanding of the workflow and the existence of variation in the care processes and revealing the possible barriers and facilitators to adhere to protocols is needed. Especially, to start the dialogue between healthcare professionals about whether this variation is desirable or not with regard to patient safety. Actions can then be taken to align WAI and WAD.

The Safety-II approach should not be considered as a replacement for Safety-I, but rather as complementary to each other [20]. From the Safety-I perspective, hospitals can provide safety indicators to measure the compliance with their protocols, whereas the Safety-II perspective takes into account the resilience of a healthcare system and anticipation to the variation of daily practice [20]. This is the first large-scale study to examine patient safety in Dutch hospitals by combining the Safety-I and Safety-II perspectives.

## Research objectives

The primary objective is to determine the effects of the FRAM intervention on the following quantitative outcomes: compliance with safety indicators and employee resilience among nurses. The safety indicators are collected for three patient safety themes: risk screening of frail older patients, the administration of high-risk medication and performing medication reconciliation at discharge.

The secondary objective is to reveal possible discrepancies between hospital protocols (WAI) and clinical practice (WAD) for the patient safety themes mentioned above. This could explain when and why healthcare professionals have to adjust their performance.

The third objective is to understand how insight into the discrepancies between practice and protocol assists healthcare providers to bridge the gap between protocols and daily practice by aiming for behavioural and/or organisational change to improve patient safety.

## Methods

### Patient Safety themes

The FRAM will be applied to three patient safety themes: risk screening of frail older patients, the administration of high-risk medication, and performing medication reconciliation at discharge. These themes were part of the Dutch Safety Management System (VMS) [3, 11, 12, 30].

Older patients are more likely to experience adverse events and are at a greater risk of developing complications causing loss of function during hospitalisation [9, 31, 32]. A screening tool that is mandatory to use in Dutch hospitals to recognise frail patients is the VMS frailty questionnaire [30, 31, 33]. The VMS frailty questionnaire consists of 13 questions and aims to identify patients aged 70 years or older at risk for malnutrition (Short Nutritional Assessment Questionnaire (SNAQ) or the Malnutrition Universal Screening Tool (MUST)) [34, 35], delirium (assessed with three questions about memory problems, previous delirium and help with Activities of Daily Living (ADL) [31]), physical limitations (scored with the 6 item Katz-index on independence in ADL [36]) and falls (assessed with a single question about whether the patient has had a fall in the past 6 months) in order to take appropriate measures to prevent/treat these problems [31]. The instrument is developed based on literature and expert opinion. Frailty is indicated as having an increased risk of suffering from one or more of these problems [37].

Secondly, we will look at high-risk medication which is applicable to 90% of all patients during hospitalisation [38]. Since 2009, a protocol for administering high-risk medication comprising 25 proceedings is used. This protocol includes conducting a double check, defined as: ‘*a procedure that requires two qualified healthcare professionals, usually nurses, who independently check the medication before administration to patients*’ [39, 40]. Compliance with the double check in practice varies from 45 to 90% [24, 39, 41–43]. One recent study showed the double check is conducted correctly in only 47% of administrations [43]. Staff shortages and time constraints are reported as the main reasons for noncompliance [39, 43].

Medication reconciliation is important for decreasing errors on discharge and during transitions of care [44, 45]. It entails compiling the most complete and accurate list of all the patient’s medications and comparing this to the physician’s admission, transfer and/or discharge prescriptions [46]. It comprises:

1. Verify (collect current medication list);
2. Clarify (make sure the medications and doses are appropriate);
3. Reconcile (compare new medications with the list and document changes in the prescriptions for medication);
4. Transfer (communicate the updated and verified list to the appropriate healthcare providers and to the patient) [47, 48].

A recent study showed that in only 44% of the examined cases, all four steps of medication reconciliation were performed correctly [49]. Medication reconciliation is performed both on admission and discharge. This study will focus on discharge, because patients are more prone to medication discrepancies then and because the admission process is implemented quite well already [49–51].

### Design

**Fig. 1:**
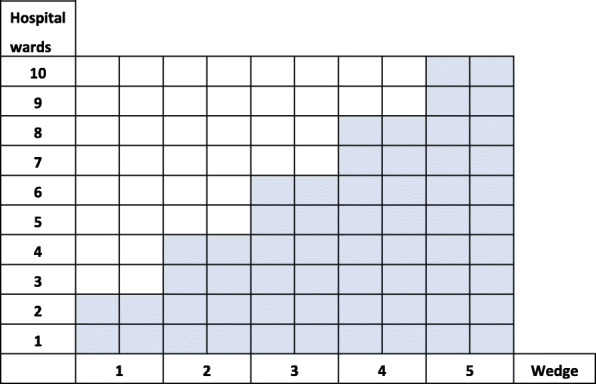
Randomisation of the wards in the stepped wedge trial

In this mixed method study, we will use a cluster randomized stepped wedge design in which every participating hospital ward will gradually receive the FRAM intervention [52–54]. This design does not deprive any ward from receiving the FRAM intervention [55, 56], while we are still able to compare data between two groups: wards that already received the FRAM intervention and those that have not yet. The study will include five wedges, each one lasting 2 months. During each wedge, two wards will receive the intervention per theme (see Fig. 1). The FRAM will be applied to all wards randomly [57]. The randomization will be carried out by an external statistician. Two criteria will be applied to the randomisation: 1) hospitals participating with more than one ward in the same theme will receive the intervention in the same wedge; 2) wards are able to indicate time periods during which they are unable to participate due to audits or shortage of staff. Patients or the public are not involved in the design, or conduct, or reporting, or dissemination plans of our research.

### Recruitment of wards

The aim is to include 10 hospital wards per theme. Although all Dutch hospitals (*N* = 74) will be invited to participate in this study, we expect only about 10 wards per theme to be interested to participate in this study due to the time investment. Therefore, the variation in type of hospitals depends on the willingness of wards to participate, and for that reason we did not apply any stratification. The inclusion criteria are shown in Table 1 and differ for each theme. Internal medicine, surgical and geriatric wards will be suitable for the theme risk screening of frail older patients because of the high number of older patients they admit. Intensive Care units (ICUs), internal medicine and surgical wards will be selected for the theme high-risk medication because they regularly administer this type of medication. Lastly, orthopaedics and cardiology wards will be selected for the theme medication reconciliation at discharge because of the high turnover of patients. Variation in university, general and teaching hospitals will depend on the willingness of hospitals to participate in the study.

**Table 1:** Eligible wards per theme

Theme	Eligible wards
Risk screening of frail older patients	Internal medicine
	Surgical wards
	Geriatric wards
	Wards with a similar patient group
High-risk medication	Intensive care units (ICUs)
	Internal medicine
	Surgical wards
Medication reconciliation	Orthopaedics
	Cardiology

**Fig. 2:**
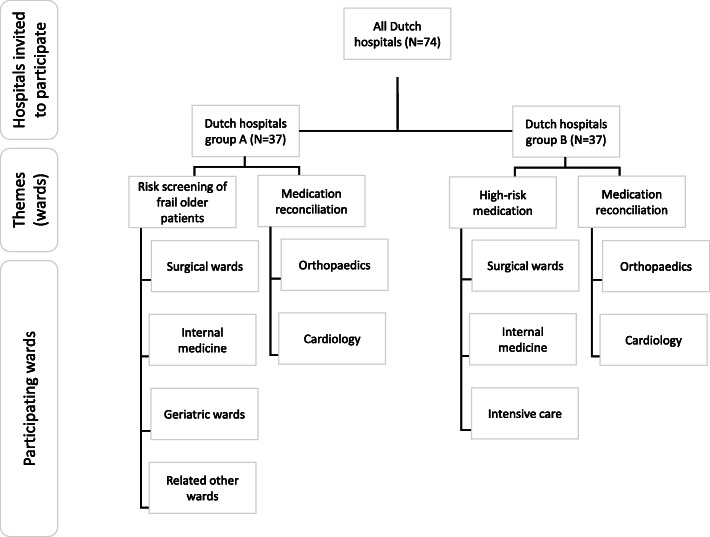
Flowchart of the recruitment of the wards

The themes risk screening of frail older patients and high-risk medication will both include the same type of wards (i.e. surgical and internal medicine). However, a ward can only participate in one of these themes to avoid a learning effect. Therefore, a list of all Dutch hospitals (*N* = 74) will be randomly divided into two groups (*N* = 37). The boards of directors will receive an invitation to participate in the studies on medication reconciliation and frail older patients, or medication reconciliation and high-risk medication (see Fig. 2). Hospitals can participate in one, both, or neither themes. The researchers will contact interested hospitals to explain the study aim and to check whether they can afford the time investment. Hospitals are not eligible if undergoing changes such as mergers or the implementation of electronic health records during data collection. To enrol in the study, a signed informed consent form will be required (see Appendix A).

### FRAM intervention

**Fig. 3:**
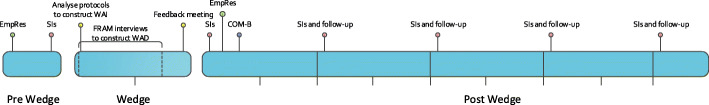
Overview of the data collection activities for each ward. The line outlines each wedge in the stepped wedge trial. Safety Indicators are shown as SIs

The FRAM intervention consists of constructing FRAM models and discussing the discrepancies between WAI and WAD with each ward in a feedback meeting. Data will be collected over 16 months in 2020 and 2021 at different points in time before, during, and after a ward is enrolled in the wedge (see Fig. 3).

#### Constructing FRAM models

The FRAM allows all activities within selected processes to be visualised in a hexagon, showing how they relate to each other and how they interact [21]. This can be used to understand how procedures work in the real world. The method is based on six aspects [29, 58]:

1. **I**nput: what starts or changes the function.
2. **O**utput: the outcome of the function.
3. **P**re-condition: conditions that need to be fulfilled to perform the function.
4. **R**esources: what the function needs or consumes.
5. **T**ime: aspects of time that affect the function.
6. **C**ontrol: factors that influence or adjust the function.

Firstly, the WAI model will be described based upon the hospital protocols. Secondly, according to the FRAM, a WAD is drawn up on the basis of interviews with healthcare professionals involved in the care processes [58]. Therefore, semi-structured interviews will be held with eight involved healthcare professionals on the ward participating such as nurses, doctors and pharmacy technicians [59]. These healthcare professionals will be recruited by the contact person of the ward, based on availability. The interviews, based on the FRAM aspects, will take approximately 30 min. Participation in the FRAM interviews will be voluntary and information will be processed anonymously.

#### Feedback meeting

A feedback meeting will be held with healthcare professionals from the participating wards to discuss any discrepancies between the WAI and WAD and, if necessary, to further complete the WAD. This should reveal where and why practice variation arises and whether this variation is desired in order to fulfil the patients’ needs better and to ensure patient safety [59]. Furthermore, it may reveal the barriers and facilitators in daily practice when adhering to the WAI. The researcher will introduce the FRAM and present the findings from the interviews. Gaining insight into the activities where variation arises could be instructive and result in organisational learning. The ward will be asked to fill out a plan of action with suggestions for aligning WAI and WAD better.

### Quantitative outcome assessments

#### Safety indicators

Performance on safety indicators, developed during the VMS programme, will be collected to monitor compliance rates. Table 2 shows which indicators will be collected for each theme. These will be used as a proxy for measuring whether care has been registered according to the protocols. From January 2020 until April 2021 the contact person on the wards of those participating in the themes about risk screening of frail older patients and medication reconciliation at discharge, will provide monthly data about the indicators. For these themes, indicators can be extracted from the electronic health record. However, this is not possible for the administration of high-risk medication, which needs to be registered in order to continue in the electronic health record. In most hospitals the registration will be 100%, even if the double check is not conducted according to the protocol. We will, therefore, ask the interviewees to register how often the double check was conducted and what the reasons were if the double check could not be conducted correctly.

**Table 2:** Collected Safety Indicators for each theme

Risk screening of frail older patients	High-risk medication	Medication reconciliation
- Percentage of patients aged ≥70 years admitted who are screened for frailty within 24 h after admission with the indicators, malnutrition, delirium, physical limitations, and fall risks. - Percentage of patients aged ≥70 years admitted who are screened within 24 h after admission with each individual indicator for: ° malnutrition ° delirium ° physical limitations ° falls.	- Percentage of high-risk medication administered where the double check was correctly conducted (self-reported).	- Percentage of discharged patients aged ≥18 years where medication reconciliation was performed at discharge.

#### Employee resilience

We will use the Employee Resilience Scale (EmpRes) to measure the effect of the FRAM intervention on employee resilience among nurses who work at the participating wards. The online questionnaire will be distributed once the ward enrols in the FRAM intervention and at the end of the wedge. The most recent version of the EmpRes scale will be used which includes nine items [60] based on elements such as: ‘learning orientation’, ‘proactive posture’, ‘positive outlook’, ‘network leveraging’ and ‘adaptive capacity’ [61]. Three changes were made to the existing questionnaire involving language and cultural changes and adding questions about team resilience to the existing questions about personal resilience. These changes and the final questionnaire can be found in Appendix B.

### Qualitative outcome assessments

#### COM-B interview

Approximately 4 weeks after the feedback meeting, the contact person on the participating ward (e.g. head of the ward/head of nurse), will be interviewed for approximately 20 min about whether the FRAM intervention has led to any intended or implemented behavioural or organisational changes. This person does not have to be responsible for implementing changes during this study, but has an overview of the ward and can therefore report back on the current status. If healthcare professionals understand the care processes better after results are presented during the feedback meeting, it may result in proposed behavioural changes to be able to improve care. Sometimes, it may also be necessary to change the flow of processes or organisation policies. The COM-B model will be used as a framework to understand these behavioural changes [62]. This includes three conditions for behavioural change: capability, opportunity, and motivation. The interview questions will be based on these components.

#### Follow-up interviews

Approximately 2 months after wards have participated in the FRAM-intervention, the ward contact persons will be asked about the implementation of changes resulting from discussing the discrepancies between WAI and WAD. The timing of these follow-ups interviews depends on the moment of enrolment. There may be practical changes in care processes or in protocols to align WAI and WAD better. We will give a qualitative description of the changes that wards have implemented or are planning to implement.

#### Sample size

For the first quantitative outcome, measuring compliance with hospital protocols, we will use all available data from participating wards. For the second quantitative outcome, measuring resilience among nurses, we aim at a response rate of 50% on the Employee Resilience Scale [63, 64]. We cannot perform a sample size calculation, since we believe that they depend on assumptions and estimates which are less suitable for smaller, pragmatic research. Within the available time, we will include the maximum sample size that is reasonably feasible, depending on the willingness of hospital wards to participate in this study [65].

For the FRAM interviews, we aim to reach data saturation by including at least 10 hospital wards for each theme (30 wards in total). We expect to reach data saturation after conducting approximately eight interviews with involved healthcare professionals (e.g. physicians, nurses, pharmacists (assistants)) per ward [22]. However, since our outcome measure regarding the FRAM intervention (i.e. revealing discrepancies between WAI and WAD and understanding practice variation) is qualitative, we will not use a sample calculation based on this outcome.

## Ethics

The study has been assessed by the Medical Ethics Committee of the VU University Medical Center Amsterdam and they have declared that the study is not subjected to Medical Scientific Research with humans (WMO) (number 2019.571). Verbal informed consent will be obtained from all participants who take part in the study at the beginning of each interview. The interviews will be recorded, transcribed and then coded, so that data cannot be traced back to the participants. Data will be stored in a secure database, accessible only to the research team.

### Data analysis

#### Safety indicators

The percentages of the safety indicators, plus the self-reported data for high-risk medication, will provide information about the compliance rates with the indicators. For data analysis, a linear mixed model with a random effect for cluster and a fixed effect for time will be used as set out by Hussey et al. [66].

#### Employee resilience

The data collected on individual and team levels of resilience will be analysed on ward level and described at an aggregated level for all wards combined. The averages of all the answers given per question will be used to analyse the data. A Wilcoxon signed rank test will be conducted in STATA (version SE 15.0) to examine the difference between the matched-paired questions [67].

#### Interviews

All interviews will be audio recorded, transcribed and a membercheck will be sent to the respondent. For the FRAM interviews, the researchers will individually distil the activities in each care process and treat them as themes using a deductive approach based on thematic analysis framework of Braun & Clarke [68]. The data will then be coded in MAXQDA to identify all relevant aspects related to the FRAM (input, output, precondition, resource, control, time) and determine the variability of each function. After coding the interviews, the WAD per ward will be made visual using the programme FRAM model Visualiser Pro [69].

The COM-B interviews will be open coded, based on the COM-B model, using MAXQDA by two researchers independently.

For both FRAM interviews and COM-B interviews, the researchers will double code 20% of the transcripts for each theme to reach consensus on how to achieve a coding scheme for all three patient safety themes. After consensus is reached, we will use axial coding to compare and link all the open codes [70]. By selective coding, we will structure, further, the plurality of the codes and the sub-codes. A qualitative description of the analysed data will be given, for example by categorizing the organisational or behavioural changes.

From the follow-up interviews we will distil categories, for example adjustments in protocols and behavioural changes, that hospital wards are planning to implement or have implemented. The purpose of these interviews is to gain insight into the changes that have taken place or going to take place and the frequency of these changes.

## Discussion

This is a study protocol for a stepped wedge study with the primary objective to examine the potential use of the FRAM as an intervention tool to improve patient safety by revealing possible discrepancies between hospital protocols (WAI) and daily practice (WAD). Based on the FRAM interviews and the following discussions with participating healthcare teams, we aim to assess variation in daily practice and determine at which points professionals deliberately, or accidentally, vary from guidelines and protocols. Furthermore, it may reveal the barriers and facilitators in the conduct of each healthcare process investigated.

### Strengths and limitations

The first strength of this study is the scope of the study population (potential to recruit across the Netherlands). The second strength is the mixed method approach. Combining the Safety-I and Safety-II approach, allows us to thoroughly examine patient safety from different perspectives. The increased compliance derived from the Safety-I approach may help to improve patient safety by identifying the failure of individuals and/or systems [20, 21]. However, argued from the Safety-II perspective, compliance rates may not do justice to the complexity of clinical practice. There could be a lack of conformity between daily practice and how the process is described in guidelines and protocols. This is simply due to the adaptability of professionals. Insight into how the care process takes place in practice - the Safety-II perspective - could increase an understanding of the three areas of care we examine. This may lead to suggestions on how to better align WAI and WAD [71]. By making protocols more practicable, they become easier for professionals to comply with and to discuss which variation is undesirable (Safety-II perspective). If WAD is more in line with WAI, this may also improve the registration of the safety indicators (Safety-I perspective). Therefore, it is desirable to look beyond the rates of compliance and use the Safety-II perspective to understand how protocols work in the real world. This could then lead to opportunities for organizational learning [24]. Third, by applying the FRAM to three different healthcare processes, this study will indicate whether it may be useful, and feasible, for hospitals to use it for analysing various healthcare processes. The detailed description of how to apply the FRAM makes it easier for hospitals to use the FRAM themselves.

There are also some limitations. Firstly, the study will be conducted on selected wards which may limit the generalizability of the results to other wards. However, we believe that the main conclusions of our multi-disciplinary study will be generally valid as wards are selected from both academic and general hospitals. Secondly, the FRAM may not be easy to apply in large complex systems which are constantly exposed to pressures for quality, safety, and productivity, and which are, at the same time, subjected to time pressures and scarcities of personnel and equipment [59, 72]. It may be hard to construct a FRAM model for complex processes, especially if boundaries are not properly defined. In such cases, the visual representation of the healthcare processes selected can become overwhelming and difficult to interpret [59]. Thirdly, even though the intervention period within each ward will only last 2 months, the total data collection period will be 16 months. Due to the stepped wedge design, it might be challenging to keep all participating wards engaged in the study before and after the intervention. For some wards there may be a long period of time between recruitment and the moment the ward receives the intervention. While, for others that receive the intervention in the first wedge(s), there will be some waiting time until the last follow up measure. We aim to keep all wards engaged in the study by collecting the safety indicators monthly and monitoring which changes they make following the intervention.

## Supplementary Information

**Additional file 1.**

**Additional file 2.**

## Data Availability

All principal investigators (LMvD and MDM) will be given access to the cleaned data sets. To ensure confidentiality, data dispersed to project team members will be blinded of any identifying participant information. Anonymized trial data will be available for non-commercial research purposes only upon request to the principal investigators (LMvD and MDM). All results will be reported to the client and will be publicly available through the Nivel website. Topics suggested for presentation or publication will be circulated to the principal investigators (LMvD and MDM). The principal investigators (LMvD and MDM) of an ancillary study should be considered as the lead authors of material derived from this study.

## Abbreviations

ADL: Activities in Daily Living
AEs: Adverse events
EmpRes: Employee Resilience Scale
VMS: Dutch National Safety Program
ICUs: Intensive Care Units
FRAM: Functional Resonance Analysis Method
WMO: Medical Scientific Research with humans
MUST: Malnutrition Universal Screening Tool
VMS: Safety Management System
SNAQ: Short Nutritional Assessment Questionnaire
WAD: Work-As-Done
WAI: Work-As-Imagined
WHO: World Health Organisation
