# Supplementary material for: Can using the functional resonance analysis method, as an intervention, improve patient safety in hospitals?: a stepped wedge design protocol
Source: BMC Health Serv Res. 2021 Nov 13;21:1228. doi: 10.1186/s12913-021-07244-z (PMC8590349; doi:10.1186/s12913-021-07244-z)
Supplement: Supplementary file 1 — Additional file 1. [file 12913_2021_7244_MOESM1_ESM.doc]

Appendix A: Informed consent form (Dutch and English)

*Original form in Dutch*

Verklaring onderzoek deelname aan het onderzoek: Monitor patiëntveiligheid 2019-2022: Derde evaluatie van VMS thema’s in Nederlandse ziekenhuizen - een Safety-II benadering met behulp van een FRAM procesanalyse.

Ondergetekende verklaart hierbij dat **<naam ziekenhuis>**

1. Onderzoekers van het NIVEL en APH instituut opdracht geeft de ‘Monitor patiëntveiligheid 2019-2022: Derde evaluatie van VMS thema’s in Nederlandse ziekenhuizen - een Safety-II benadering met behulp van een FRAM procesanalyse’ uit te voeren in het ziekenhuis.
2. **Wel / Niet** (doorhalen wat niet van toepassing is) als deelnemer vernoemd wil worden in het dankwoord van de rapportage over de uitkomsten van het onderzoek (de individuele resultaten van uw ziekenhuis zullen ten allen tijde niet openbaar gerapporteerd worden).

Naam:…………………………………………………………………………….

Functie:…………………………………………………………………………...

Datum:……………………………………………………………………………

Handtekening:……………………………………………………………………

*English translation*

Consent participation in the research: Patient safety monitor 2019-2022: Third evaluation of patient safety themes in Dutch hospitals – a Safety-II approach using FRAM analysis.

The undersigned, hereby declares that <**hospital name>**

1. Authorizes researchers from NIVEL and APH institute to conduct the ‘Patient safety monitor 2019-2022: Third evaluation of patient safety themes in Dutch hospitals – a Safety-II approach using FRAM analysis’ in this hospital.
2. **Does / Does not** (cross out what does not apply) want to be named as a participant in the acknowledgements in the report on the outcomes of the research (the individual results from your hospital will not be reported publicly).

Name:…………………………………………………………………………….

Job title:…………………………………………………………………………...

Date:……………………………………………………………………………

Autograph:……………………………………………………………………
