# Supplementary material for: Can using the functional resonance analysis method, as an intervention, improve patient safety in hospitals?: a stepped wedge design protocol
Source: BMC Health Serv Res. 2021 Nov 13;21:1228. doi: 10.1186/s12913-021-07244-z (PMC8590349; doi:10.1186/s12913-021-07244-z)
Supplement: Supplementary file 2 — Additional file 2. [file 12913_2021_7244_MOESM2_ESM.docx]

# Appendix B: Employee resilience scale (EmpRes)

As mentioned, three changes were made to the existing questionnaire. Firstly, the questions were translated backwards and forward from English to Dutch, following the stages of cross-cultural adaptation recommendations. Using the stages of cross- cultural adaptation recommendations can help in looking both at language and cultural issues when using a questionnaire in a different setting. The questions were first translated to Dutch by two translators (stage I). After the two translators reached a consensus (stage II), the items were translated back into English by native speakers (stage III) to ensure that the questions had been translated correctly. The adapted questionnaire was then reviewed by experts (stage IV) and test interviews with nurses were held to find out how questions were interpreted (stage V). Secondly, questions about team resilience were added as the original ones were based solely on personal resilience and may lead to answers which are socially desirable. Results at team level can complement the picture. Thirdly, two propositions were added asking, how often the respondent was able to fulfil, successfully, their role and what considerations they make regarding screening for frailty, performing medication reconciliation, or administering high-risk medication. We also asked the respondents for their job title and years of experience in their current position.

## **EmpRes questionnaire**

| Dutch | English |
| --- | --- |
| Ik werk effectief samen met anderen om met onverwachte uitdagingen op mijn werk om te gaan. | I effectively collaborate with others to handle unexpected challenges at work. |
| Ik slaag erin om voor langere periodes met een hoge werkdruk om te gaan. | I successfully manage a high workload for long periods of time. |
| Ik los een crisis op het werk competent op. | I resolve crises competently at work. |
| Ik leer van de fouten op het werk en verbeter de manier waarop ik mijn werk doe. | I learn from mistakes at work and improve the way I do my job. |
| Ik evalueer mijn prestaties en verbeter continu mijn manier van werken | I re-evaluate my performance and continually improve the way I do my work. |
| Ik reageer effectief op feedback die ik op het werk krijg, zelfs op kritiek. | I effectively respond to feedback at work, even criticism. |
| Ik zoek naar ondersteuning bij mijn werk indien ik specifieke hulp of hulpmiddelen nodig heb. | I seek assistance to work when I need specific resources. |
| Ik benader een manager / supervisor / leidinggevende wanneer ik hun ondersteuning nodig heb. | I approach managers when I need their support. |
| Ik gebruik verandering op het werk als mogelijkheid om te groeien. | I use change at work as an opportunity for growth. |

Table 1: EmpRes questionnaire in Dutch and English
